# International proficiency trial demonstrates reliable Schmallenberg virus infection diagnosis in endemic and non-affected countries

**DOI:** 10.1371/journal.pone.0219054

**Published:** 2019-06-27

**Authors:** Kerstin Wernike, Martin Beer

**Affiliations:** Institute of Diagnostic Virology, Friedrich-Loeffler-Institut (FLI), Greifswald—Insel Riems, Germany; Faculty of Science, Ain Shams University (ASU), EGYPT

## Abstract

Schmallenberg virus (SBV), an orthobunyavirus infecting ruminants, emerged in 2011 in Central Europe, spread very rapidly throughout the continent and established an endemic status, thereby representing a constant threat not only to the European livestock population, but also to neighboring countries. Hence, in endemically infected regions, the maintenance and regular verification of diagnostics is needed and in not yet affected regions, suitable diagnostic systems should be established to be prepared for a potential introduction of the disease. In addition, also for the trade of animals into free regions, highly reliable and sensitive diagnostics are of utmost importance. Therefore, a laboratory proficiency trial was initiated to allow for performance evaluations of test systems available for SBV-diagnostics, but also for evaluation of veterinary diagnostic laboratories performing those tests. Ten serum samples (six seropositive, four seronegative) were provided for serological analysis, four of the seropositive samples were provided undiluted, while the remaining samples represented 1/2 and 1/4 dilutions of one of the aforementioned samples in negative serum. Ten further sera (five virus-positive, five negative) were sent to the participants to be analyzed by SBV genome detection methods. A total of 48 diagnostic laboratories from 15 countries of three continents (Europe, Asia, North America) and three kit manufacturers participated in the SBV proficiency test, thereby generating 131 result sets, corresponding to 1310 individual results. The sample panel aimed for serological analysis was tested 72 times; the applied diagnostic methods comprised different commercial ELISAs and standard micro-neutralization tests. The sample set aimed for genome detection was analyzed in 59 approaches by various commercial or in-house (real-time) RT-PCR protocols. Antibody or genome positive samples were correctly identified in every case, independent of the applied diagnostic test system. For seronegative samples, three incorrect, false-positive test results were produced. Virus-negative samples tested false-positive in two cases. Thus, a very high diagnostic accuracy of 99.58% and 99.66% was achieved by the serological and virological methods, respectively. Hence, this ring trial demonstrated that reliable and robust SBV-diagnostics has been established in veterinary diagnostic laboratories in affected and non-affected countries.

## Introduction

Schmallenberg virus (SBV), a member of the genus *Orthobunyavirus* (order *Bunyavirales*), infects predominantly ruminants, thereby inducing a short-lived viremia of two to six days, in cattle sometimes associated with a mild, transient disease characterized by fever, diarrhea and decreased milk production [1, 2]. However, when pregnant cattle or ewes are infected during a critical period of gestation, SBV may cross the placental barrier and infect the fetus occasionally leading to abortion and/or severe fetal malformation summarized as arthrogryposis-hydranencephaly syndrome [3–5]. Between its mammalian hosts, SBV is transmitted by *Culicoides* biting midges of various species [6–10].

SBV initially emerged in 2011 in the German/Dutch border region [1]. Thereafter, it spread rapidly throughout the European continent reaching the Scandinavian countries and the British Isles at the North, the Mediterranean region including Spain, Southern France, Italy, Greece and Turkey at the South and Eastern European countries such as Poland or Lithuania and Russia [11–21]. In subsequent years, alternating low-level circulation and re-emergence to a larger extend have been reported from affected regions [22–26]. Therefore, it has to be anticipated that SBV has established an enzootic status in Europe and will re-appear to a larger extent in regular intervals in the future, especially since such patterns of cyclic re-emergence are well-known from closely related viruses [27–30]. Thus, the maintenance and constant verification of reliable diagnostics is needed. Furthermore, diagnostic systems should be established and regularly evaluated in not yet affected countries bordering the endemically infected regions, since a further spread of the disease cannot be excluded, particularly because the insect vectors responsible for virus transmission are wide-spread [31, 32].

The direct detection of SBV is primarily based on RT-PCR systems, either in the form of different commercially available real-time RT-PCR kits or various in-house real-time or conventional RT-PCR protocols (e.g., [1, 33, 34]. In adult animals, the preferred sample material is serum. However, the direct detection of virus or viral genome is time restricted by the short-lived viremia of only a few days. Therefore, the detection of specific antibodies, which are induced between one and three weeks after infection [2, 35–37] and persist for several years [38–40], is more promising for SBV-infection diagnosis. For serological analysis, several commercial or in-house ELISAs, micro-neutralization or indirect immunofluorescence tests are available [36, 41–43].

Here, test systems routinely used for SBV-infection diagnostics have been evaluated in the context of an international interlaboratory proficiency trial. A panel of standardized samples was sent to veterinary diagnostic laboratories across the world with the request to analyze the samples by serological and virological methods routinely applied in the respective institution.

## Materials and methods

A total of 20 serum samples were provided, where 10 samples were aimed for the detection of viral genome, and 10 samples for serological analysis. Aliquots of 1ml were prepared in 2-ml injection bottles (Zscheile & Klinger GmbH, Hamburg, Germany) and lyophilized. The bottles were subsequently sealed with rubber plug and flanged caps (both Zscheile & Klinger GmbH) and stored at 4°C until sent to the participating institutions.

The sample panel for serological analysis included two sheep and two cattle sera negative for antibodies against SBV, and six antibody positive sera (1x sheep, 5x cattle), which were collected 3, 4, or 12 weeks after experimental SBV-infection [2, 36, 44]. The sample SBV-S-8 represented a 1/2 dilution of sample SBV-S-10 in SBV antibody negative serum and the sample SBV-S-1 represented a 1/4 dilution of sample SBV-S-10 (Table 1).

**Table 1:** Status of the samples sent for Schmallenberg virus (SBV) infection diagnosis to the ring trial participants. The results of the pre-testing by real-time RT-PCR [33] or microneutralization test [36] prior to shipment and the time points at which the samples for viral genome detection were taken after experimental infection are given in parenthesis. Cq–quantification cycle value, dpi–days post infection.

ring trial number	animal species	sample status (neutralizing titer or Cq value)
SBV-S-1	cattle	SBV antibody positive, 1/4 dilution of sample SBV-S-10 (1/28)
SBV-S-2	sheep	SBV antibody negative (< 1/5)
SBV-S-3	cattle	SBV antibody positive (1/90)
SBV-S-4	cattle	SBV antibody negative (< 1/5)
SBV-S-5	cattle	SBV antibody positive (1/28)
SBV-S-6	sheep	SBV antibody negative (< 1/5)
SBV-S-7	cattle	SBV antibody negative (< 1/5)
SBV-S-8	cattle	SBV antibody positive, 1/2 dilution of sample SBV-S10 (1/57)
SBV-S-9	sheep	SBV antibody positive (1/22)
SBV-S-10	cattle	SBV antibody positive (1/71)
SBV-P-11	cattle	SBV genome positive (25, 3dpi)
SBV-P-12	cattle	SBV genome positive (26, 3 dpi)
SBV-P-13	sheep	SBV genome negative (no Cq)
SBV-P-14	cattle	SBV genome positive (27, 4dpi)
SBV-P-15	cattle	SBV genome negative, = SBV-S-10 (no Cq)
SBV-P-16	cattle	SBV genome positive (28, 5dpi)
SBV-P-17	sheep	SBV genome negative (no Cq)
SBV-P-18	cattle	SBV genome negative (no Cq)
SBV-P-19	cattle	SBV genome positive (25, 4dpi)
SBV-P-20	cattle	SBV genome negative (no Cq)

The panel aimed for the detection of viral genome comprised two SBV genome negative sheep sera, three SBV genome negative cattle sera (the antibody positive serum SBV-S-10 and two times fetal calf serum, Biochrom GmbH, Berlin, Germany), and five cattle sera obtained during the viremic phase after experimental infection with SBV [44].

The identifiers and classifications of all samples are given in Table 1.

A total of 48 veterinary diagnostic laboratories from 15 countries (Austria, Belgium, Canada, Denmark, Finland, France, Germany, Hungary, Ireland, Israel, Italy, Netherlands, Poland, Russia, and Switzerland) and three kit manufacturers participated in the SBV proficiency test. Every German state veterinary laboratory had been invited, and every non-German reference center that asked previously whether the authors organize SBV ring trials was allowed to participate. The participants (except the kit manufacturers) perform routine diagnostics for Simbu serogroup viruses in their country and/or pre-export or pre-import investigations.

The participants were asked to analyze the provided samples with the test systems routinely used in their institution. If results were considered “doubtful” by a participant, it was assumed that the necessary clarifications/follow-up analysis would have been initiated in practice and, therefore, that no divergent result was produced.

To assess the discriminative property of the tests, the diagnostic accuracy was calculated by taking the sensitivity and specificity into account. The sensitivity represents the ability to identify a positive sample correctly and is defined as the proportion of true positive results in a set of positive cases (calculated as: true positive results/true positives + false negatives), while the specificity of a test is its ability to determine the negative cases correctly (calculated as: true negative results/true negatives + false positives) [45]. The diagnostic accuracy was finally calculated by using the free statistical calculator MedCalc (MedCalc Software, Ostend, Belgium).

## Results

### Serology

The sample panel for serological analysis was investigated in 43 laboratories by commercially available antibody ELISA tests, in some cases several test systems were used, whereby 50 result sets were generated (= 500 individual results). The applied test systems included: (I) ID Screen Schmallenberg virus Competition Multi-species, IDvet, Grabels, France (n = 28); (II) ID Screen Schmallenberg virus Indirect Multi-species, IDvet, in either the monophasic (n = 11) or biphasic (n = 5) variant; (III) IDEXX Schmallenberg Ab Test, IDEXX Europe B.V., Hoofddorp, the Netherlands (n = 4); (IV) SVANOVIR SBV-Ab, Boehringer Ingelheim Svanova, Uppsala, Sweden (n = 2). The status of the samples was correctly identified in every case (Table 2).

**Table 2:** Results of commercially available Schmallenberg virus antibody ELISAs and of the standard microneutralization tests performed by the participating laboratories. The sample status is given below the respective sample identifier.

test system		SBV-S-1 (pos)	SBV-S-2 (neg)	SBV-S-3 (pos)	SBV-S-4 (neg)	SBV-S-5 (pos)	SBV-S-6 (neg)	SBV-S-7 (neg)	SBV-S-8 (pos)	SBV-S-9 (pos)	SBV-S-10 (pos)
ID Screen Schmallenberg virus Competition Multi-species, ID.vet	no. tests	28	28	28	28	28	28	28	28	28	28
	no. positive	28	0	28	0	28	0	0	28	28	28
ID Screen Schmallenberg virus Indirect Multi-species (biph.), ID.vet	no. tests	5	5	5	5	5	5	5	5	5	5
	no. positive	5	0	5	0	5	0	0	5	5	5
ID Screen Schmallenberg virus Indirect Multi-species (monoph.), ID.vet	no. tests	11	11	11	11	11	11	11	11	11	11
	no. positive	11	0	11	0	11	0	0	11	11	11
IDEXX Schmallenberg Ab Test, IDEXX	no. tests	4	4	4	4	4	4	4	4	4	4
	no. positive	4	0	4	0	4	0	0	4	4	4
SVANOVIR SBV-Ab, Svanova	no. tests	2	2	2	2	2	2	2	2	2	2
	no. positive	2	0	2	0	2	0	0	2	2	2
microneutralization test	no. tests	22	22	22	22	22	22	22	22	22	22
	no. positive	22	2	22	0	22	1	0	22	22	22
	no. doubtful	0	4	0	0	0	1	1	0	0	0

**Fig 1:**
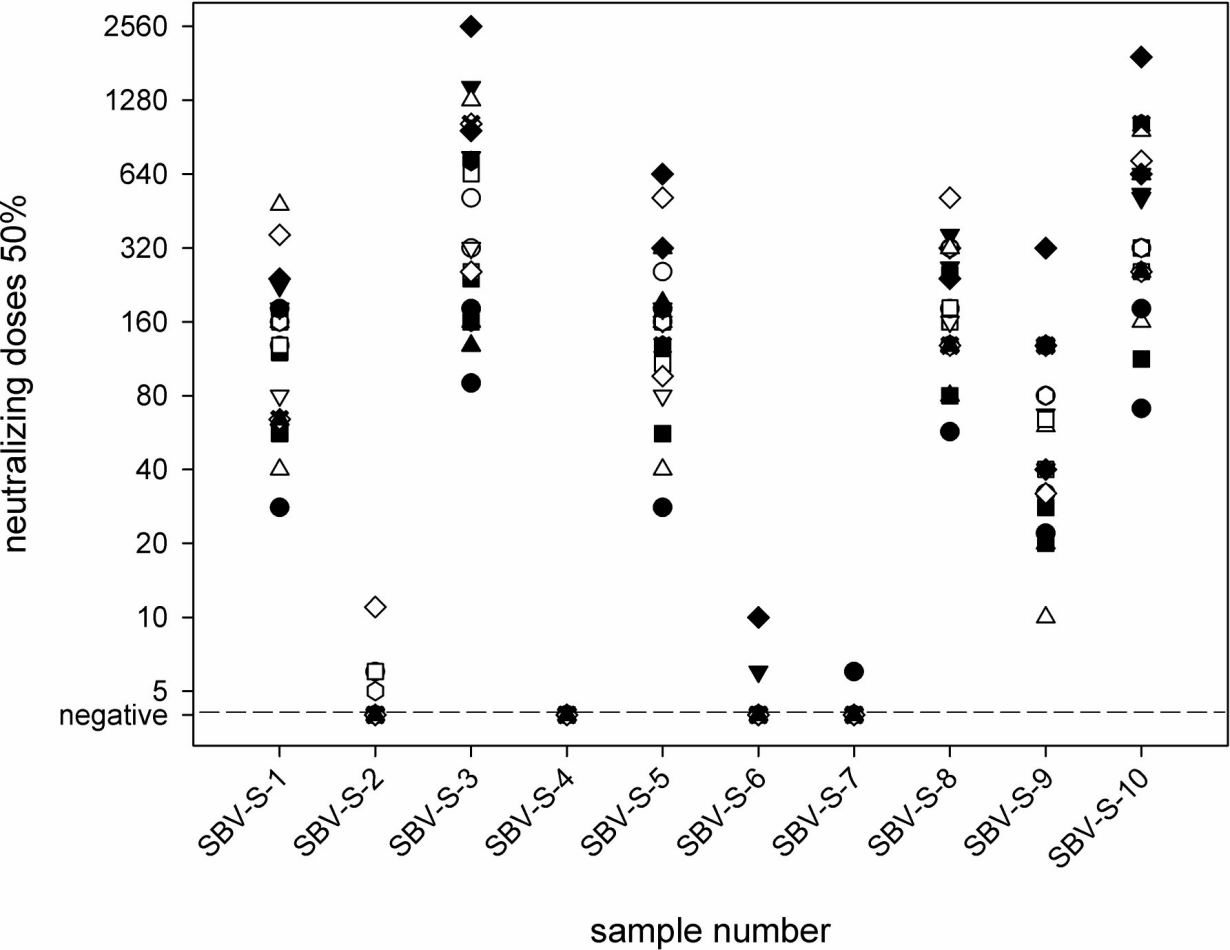
Results of the standard microneutralization tests for the serological panel. All results of a particular participant are depicted by the identical symbol for each sample.

In 22 laboratories, the samples were investigated by standard microneutralization tests in addition or alternatively to the analysis by antibody ELISA. The test was conducted in 17 laboratories according to a previously published protocol [36] using the first SBV-isolate “BH80/11” and a baby hamster kidney (BHK) cell line, and the cytopathogenic effect was assessed after two, three, four or five days. In two further cases, a BHK cell line was used as well, but the specific SBV strain was not given in the results sheet. In the remaining three laboratories, an African green monkey (Vero) cell line was used in combination with a local field strain; the cytopathogenic effect was assessed after four days. Sera containing SBV-specific antibodies (SBV-S-1, SBV-S-3, SBV-S-5, SBV-S-8, SBV-S-9, and SBV-S-10) tested positive as expected in every case, however, the resulting neutralizing titers differed markedly between the laboratories (SBV-S-1: 1/28 to 1/480; SBV-S-3: 1/90 to 1/2560; SBV-S-5: 1/28 to 640; SBV-S-8: 1/57 to 1/513; SBV-S-9: 1/10 to 1/320; SBV-S-10: 1/71 to 1/1920) (Fig 1). Antibody negative sera (SBV-S-2, SBV-S-4, SBV-S-6, and SBV-S-7) were predominantly correctly defined as being negative. The only exceptions were sample SBV-S-2, which scored doubtful in four cases and positive in two laboratories, sample SBV-S-6 that tested once doubtful and once false-positive, and sample SBV-S-7 that tested doubtful in one case (Table 2).

Considering a total number of 720 individual results produced by serological methods (500 by ELISAs and 220 by neutralization tests) and three false-positive results, a diagnostic accuracy of 99.58% was achieved.

### Genome detection

To be used for PCR analysis, nucleic acids were extracted from the provided samples either manually by using six different commercial kits or TRIzol, or automated extraction based on a wide range of commercial kits was applied. The extraction kits used by the participants included (sorted alphabetically by manufacturer): (I: manual extraction) NucleoSpin RNA Virus, MACHERY-NAGEL GmbH & Co. KG, Düren, Germany; QIAamp cador Pathogen Mini, Qiagen, Hilden, Germany; QIAamp Viral RNA Mini, Qiagen; RNeasy Mini kit, Qiagen; High Pure Viral RNA Kit, Roche, Basel, Switzerland; Invisorb Spin Virus RNA Mini Kit, Stratec Molecular GmbH, Berlin, Germany; (II: automated extraction) innuPREP Virus DNA/RNA kit, Analytik Jena AG, Jena, Germany; chemagic Viral DNA/RNA Kit PerkinElmer chemagen Technologie GmbH, Baesweiler, Germany; ID Gene Mag Fast Extraction kit, IDvet; ID Gene Mag Universal Extraction kit, IDvet; NucleoMag VET kit, MACHERY-NAGEL GmbH & Co. KG; NucleoSpin RNA Virus, MACHERY-NAGEL GmbH & Co. KG; NucleoSpin Virus kit, MACHERY-NAGEL GmbH &Co. KG; QIAamp cador Pathogen Mini, Qiagen; QIAamp Viral RNA Mini, Qiagen; QIAamp Viral RNA Mini QIAcube kit, Qiagen; MagAttract 96 cador Pathogen kit, Qiagen; MagAttract Virus Mini M48 kit, Qiagen; MagNA Pure 96 DNA and Viral NA Small Volume Kit, Roche; Ribo-Sorb RNA/DNA extraction kit, Sacace Biotechnologies Srl, Caserta, Italy; LSI MagVet Universal Isolation Kit, ThermoFisher Scientific, Waltham, USA; MagMAX CORE Nucleic Acid Purification Kit, ThermoFisher Scientific; MagMAX Pathogen RNA/DNA Kit, ThermoFisher Scientific.

For subsequent PCR analyses, commercially available real-time RT-PCR kits were used in a total of 38 approaches. The applied test systems included: (I) virotype SBV RT-PCR Kit, INDICAL BIOSCIENCE GmbH, Leipzig, Germany (n = 32); (II) virellaSBV real time RT-PCR Kit, gerbion GmbH & Co. KG, Kornwestheim, Germany (n = 3); (III) ADIAVET Schmallenberg virus real time, bioMérieux, Marcy-l’Étoile, Frankreich (n = 1); (IV) ID Gene Schmallenberg Duplex, ID.vet GENETICS, Grabels, France (n = 1); (V) VetMAX Schmallenberg Virus Kit, ThermoFisher Scientific (n = 1). In addition or alternatively to the commercial kits, in-house RT-PCR assays were applied for genome detection in 21 laboratories (16x primers and probe described in [33]; 1x [46]; 3x not further specified real-time RT-PCR protocols, 1x not further specified conventional RT-PCR protocol).

**Fig 2:**
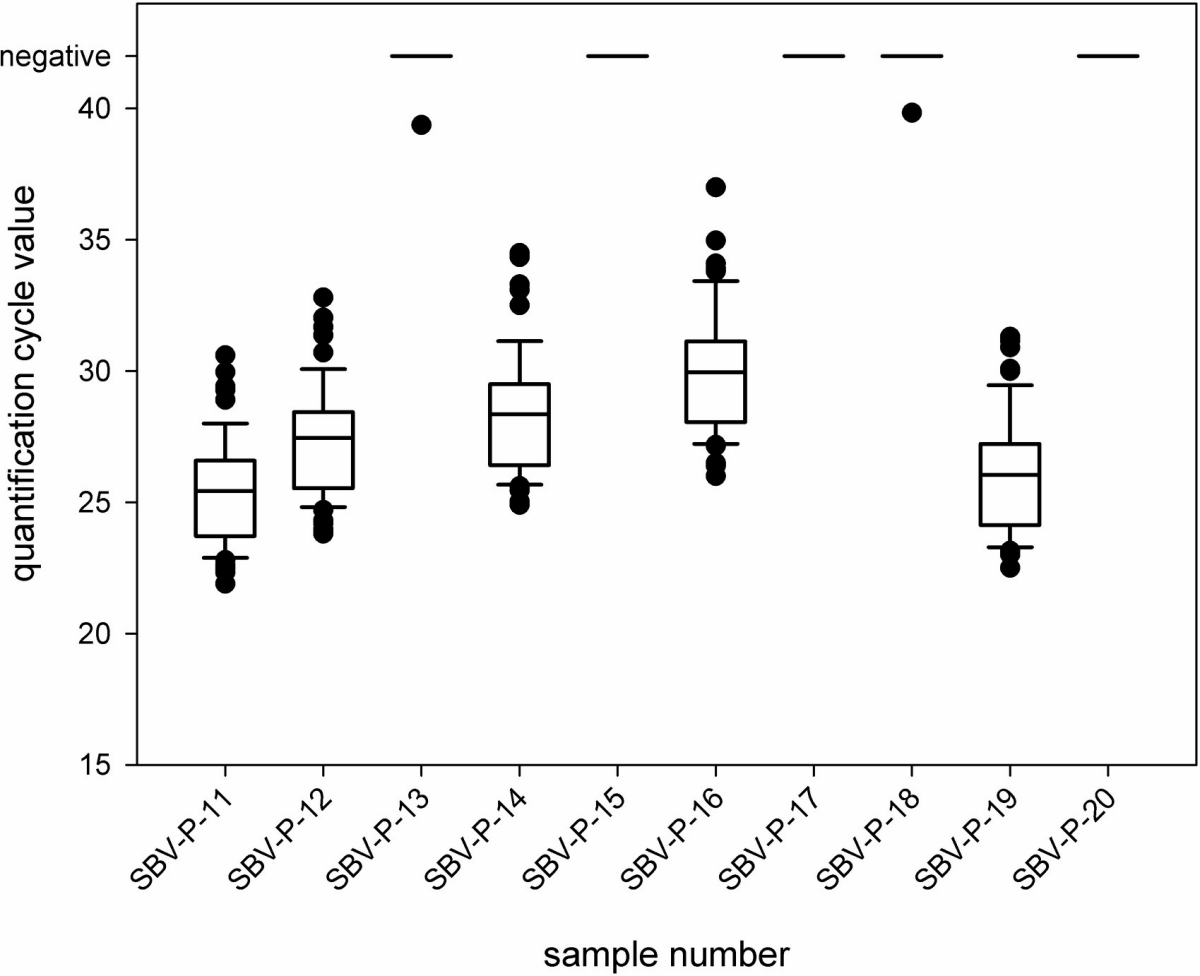
Quantification cycle (Cq) values produced by the ring trial participants using different real-time RT-PCR systems. Each outlier is depicted by a dot.

Positive samples (SBV-P-11, SBV-P-12, SBV-P-14, SBV-P-16, and SBV-P-19) were correctly identified in every case independent from the applied test system. The quantification cycle (Cq) values determined for each individual sample are shown in Fig 2 as box plots.

The negative samples (SBV-P-13, SBV-P-15, SBV-P-17, SBV-P-18, and SBV-P-20) were tested correctly negative, with the exception of samples SBV-P-13 and SBV-P-18, which tested weak positive in one case each. The Cq values of these incorrect results were 39.4 (sample SBV-P-13) and 39.8 (SBV-P-18), respectively, and therefore very close to the detection limit.

Since the sample panel for genome detection has been tested by 59 extraction/PCR approaches, thereby generating 590 individual results, and only two incorrect results were produced, the sample status was correctly identified in 99.66% of the analyses.

## Discussion

Insect-transmitted viruses have been emerging and spreading for centuries, however, their incidence and geographical spread is more rapid and extensive nowadays. Factors that were claimed to be the reasons of this phenomenon range from climate change and the intensive growth of the global transportation systems to an increase in urbanization and conversion of land to agricultural use [47]. An example for such an emerging and intensively circulating virus of veterinary importance represents SBV, which initially emerged in Central Europe and subsequently spread very rapidly throughout the European Union [21]. Thereafter, the virus established an endemic status in Central Europe, thereby presenting a constant threat to the ruminant population, which makes the maintenance of a high quality standard of SBV diagnostics highly important.

In addition, trade restrictions for live animals and bovine semen have been implemented in several non-affected countries [48], which can be circumvented by appropriate pre-export checks. This further highlights the importance of highly reliable diagnostics that should be established not only in endemically affected countries, but also in areas at risk for disease introduction.

One way of assuring the quality of diagnostics and to independently assess the quality of results produced in diagnostic laboratories represents the participation in interlaboratory proficiency trials. By ring tests, the competency of a laboratory as well as of the applied methods can be demonstrated to accreditation or other regulatory bodies [49]. That is why SBV proficiency trials have been initiated among laboratories located in the initially mostly affected regions shortly after the establishment of first methods for either the serological or virological diagnosis of SBV-infections [50, 51]. However, to ensure a high level of quality, laboratories should participate in ring trials on a regular basis, as has been accomplished in Germany, where an infection with SBV represents a reportable disease (Regulation on Reportable Animal Diseases, http://www.gesetze-im-internet.de/tkrmeldpflv_1983/BJNR010950983.html), and where ring trials are organized regularly [51, 52].

Here, we describe the assessment of the diagnostic capacity of numerous European laboratories and, for the first time, also of laboratories from further continents, i.e. Asia and North America. Serum samples taken during the viremic phase after experimental SBV-infection were provided and viral genome was reliably detected in every case regardless of the applied nucleic acid extraction/RT-PCR assay combination, which is quite considerable when one recalls the very large number of used extraction methods. In terms of specificity, five SBV-negative sera were tested 59 times and false-positive results were only generated in two cases (one incorrect test result each in two different laboratories). Possible explanations for these incorrect test results might be improper PCR conditions or unspecific reactions of the applied PCR reagents [53]. However, different extraction methods (manual vs. automated) and two distinct commercial real-time RT-PCR kits were used and the genome negative samples were tested in both cases in direct proximity to a highly positive sample. Therefore, the most likely explanation is cross-contamination during nucleic acid extraction or PCR preparation, which is a well-known risk in molecular diagnostics, especially when carried out in conjunction with the propagation and application of virus- or plasmid-based positive controls or sequencing [54, 55]. In order to notice such contamination the constant inclusion of proper controls, such as a sufficient number of negative extraction controls and no template controls, is highly recommended [53, 56], thereby preventing incorrect examination reports.

When analyzing the serological sample panel, an excellent sensitivity of 100% was achieved as well. Moreover, despite a previous study reported clear differences in the diagnostic performance of commercial SBV antibody ELISAs [57], this optimal sensitivity of 100% was reached in the present study regardless of whether a commercial kit or a cell-culture based method has been used. However, when comparing the titers measured by the applied microneutralization tests considerable variations are noticeable. This phenomenon has been already described earlier [50, 52], and is most likely caused by the variations in the test protocols of this biological assay, such as different cell lines, virus isolates or viral doses and readout after two, three, four or five days. Nonetheless, the final classifications, i.e. the identification of positive samples, was in full agreement between the participating laboratories. In terms of specificity, three false-positive results were produced leading to the questions as to whether the cut-off should be adjusted or the test protocol slightly modified in the concerned laboratories.

In conclusion, a very high overall diagnostic accuracy of 99.62% was achieved in this SBV ring trial. Hence, the presented interlaboratory proficiency test demonstrated that reliable SBV-infection diagnostics was established and maintained for both, antibody and viral genome detection.
